# Cross-sectional study of clinical and linguistic characteristics mental disorders in HIV infection

**DOI:** 10.1192/j.eurpsy.2021.665

**Published:** 2021-08-13

**Authors:** N. Neznanov, N. Khalezova, N. Burmistrova, A. Tabulina

**Affiliations:** Psychiatry And Narcology, FSBI First Pavlov Medical Univercity, Sankt Petersburg, Russian Federation

## Abstract

**Introduction:**

Current epidemiological data on the pandemic prevalence of HIV in the world expand the boundaries of the problems associated with the threat of unpredictable spread of infection and the frequency of mental disorders that accompany HIV infection.

**Objectives:**

Somatogenic mental disorders are determined by the fact that the central nervous system (CNS) is one of the reservoirs for HIV. HIV is indirectly a neurotropic virus and can cause associated neurocognitive impairment (HAND)

**Methods:**

In study were used clinical-linguistic examination method for determining linguistic markers for mental disorders in HIV patients who did not receive specific antiviral therapy.

**Results:**

As a result, it was found that 50% of HIV-infected patients not suffering from addiction syndromes or surfactant abuse have mental disorders, which are characterized mainly by disorders of adaptive reactions and mild cognitive impairment. 57.7% of HIV-infected people who do not use surfactants showed a high level of social adaptation. For patients who do not use surfactants, and who have undergone a commission examination before starting ART, the leading psychopathological syndromes are anxiety, anxiety-hypochondria. 44.2% of patients did not have obvious psychopathological symptoms.

**Conclusions:**

Structure of emotional experiences was revealed in patients who recently learned about the burden of a serious chronic disease with the corresponding fear of death and self-stigmatization. Identified linguistic markers are additional signs that can be used by physicians and psychiatrists to diagnose both cognitive impairment and emotional impairment in patients with HIV infection.

**Conflict of interest:**

No significant relationships.

